# On-line micro column preconcentration system based on amino bimodal mesoporous silica nanoparticles as a novel adsorbent for removal and speciation of chromium (III, VI) in environmental samples

**DOI:** 10.1186/s40201-015-0205-z

**Published:** 2015-05-22

**Authors:** Hamid Shirkhanloo, Aisan Khaligh, Farideh Golbabaei, Zargham Sadeghi, Amir Vahid, Alimorad Rashidi

**Affiliations:** Occupational and Environmental Health Research Center (OEHRC), Iranian Petroleum Industry Health Research Institute (IPIHRI-PIHO), Tehran, 1485733111 Iran; Research Institute of Petroleum Industry (RIPI), Tehran, 14665-1137 Iran; Department of Chemistry, Semnan University, Semnan, 35131-1911 Iran; Occupational Health Engineering Department, School of Public Health, Tehran University of Medical Sciences, Tehran, 6446-14155 Iran

**Keywords:** Chromium, Speciation, Removal, Solid phase extraction, Amine-functionalized UVM-7, Flame atomic absorption spectrometry

## Abstract

**Background:**

Chromium (VI) has toxic and carcinogenic effects. So, determination and speciation of chromium in environmental samples is very important in view of health hazards. In this study, solid phase extraction (SPE) based on bulky amine-functionalized bimodal mesoporous silica nanoparticles (NH_2_-UVM-7) as a novel nanoadsorbent was applied for preconcentration and speciation of chromium (III, VI) in water samples.

**Methods:**

UVM-7 was synthesized via atrane route and subsequently functionalized with amino silane via grafting method. In SPE procedure, polymer tubing as a micro-column was filled with NH_2_-UVM-7 adsorbent. Preconcentration and speciation of Cr (III) and Cr (VI) ions with NH_2_-UVM-7 were obtained in water samples due to the fact that only Cr (VI) ions can be complexed with-NH_2_ groups at optimized pH. Finally, chromium concentration was determined by flame atomic absorption spectrometry (F-AAS).

**Results:**

TEM, XRD, and SEM results confirmed the beneficial properties of NH_2_-UVM-7 as the adsorbent for chromium extraction. Under the optimal conditions, linear calibration curve, detection limit and preconcentration factor were obtained 6–320 μg/ L, 1.2 μg/L and 66.7, respectively (RSD < 5 %). The efficiency of nanoadsorbent for preconcentration and extraction of Cr (VI) was 96 %, whereas it was less than 5 % for Cr (III).

**Conclusions:**

The developed NH_2_-UVM7-based SPE/F-AAS method has enough sensitively and simplicity for speciation and determination of Cr (VI) and Cr (III) ions in real water samples. Good recoveries, with low detection limits and good preconcentration factors are the main advantages of this procedure.

## Background

Heavy metals in industrial and municipal effluent water (IMEW) are important sources of water contamination. The contamination and quality of IMEW are its main concern especially in the regions with limited water resources The refinery effluents are one of the most important water pollution sources which can release a large quantity of heavy metals by crude oil-processing and petrochemical industries. In such region, heavy metals such as Ni, V, Pb, Hg, Cr and Co, etc. enter into the soil, water and plants and cause environmental pollutions and poison food chain [1–4]. So, in the viewing of health risk hazards, determination of heavy metals in environmental samples is very important [5–7].

The hexavalent chromium has a serious environmental pollution and threatens human health [8]. Chromium species exist mainly in two different oxidation states in the environment, Cr (III) and Cr (VI), which have contrasting physiological effects. The Cr (III) compounds play an important role in the metabolism of glucose and certain lipids [9, 10]. On the contrary, the Cr (VI) has toxic and carcinogenic effects and damage macromolecules, proteins and DNA [11]. The World Health Organization (WHO) states that 50 μg/L of Cr (VI) are considered to be too high as compared to its gen-toxicity [11–13]. Exposure may occur from natural or industrial sources of chromium. The general population is exposed to chromium by inhaling ambient air, ingesting food, and drinking water containing chromium. WHO has set a permissible limit of 0.05 mg/L for total chromium in drinking water [14]. Thus, the accurate determination of chromium ions has become increasingly necessary to study problems associated with environmental water pollution.

In order to provide a timely warning of chromium exposure, it is highly desirable to develop suitable procedures for chromium speciation. The most sensitive analysis techniques for determination of chromium species include ion chromatography inductively coupled plasma mass spectrometry (IC-ICP-MS) [15], luminescence quenching [16], stripping voltammetry [17], flame atomic absorption spectrometry (F-AAS) [18], neutron activation analysis (NAA) [19], inductively coupled plasma optical emission spectrometry (ICP-OES) [20], isotope-dilution mass spectrometry (ID-MS) [21], and electro-thermal atomic absorption spectrometry (ET-AAS) [22, 23]. However, high instrumental and operational costs, low concentration levels of analytes, high levels of matrices, and high detection limits are common disadvantages of many of these methods. In order to simplify the analytical approaches, a separation/preconcentration step prior to analysis is required. Liquid-liquid extraction (LLE) [24, 25], homogeneous liquid-liquid extraction [26], solid phase extraction (SPE) [27, 28], liquid-phase micro-extraction (LPME) [29], and cloud point extraction (CPE) [30], etc., have been used for the preconcentration and/or separation of trace and ultra-trace amounts of chromium.

Among variety of methods, SPE is widely applied for the trace metal ions preconcentration because of its advantages including simplicity, minimal cost, rapidity, safety and ease of automation, lesser waste generation, lesser matrix effect, availability and easy recovery, high preconcentration efficiency, and easy adaptation of solid phase in a mini column coupled to a continuous flow preconcentration system [31]. In the SPE procedure, the choice of the appropriate sorbent is a critical factor to obtain full recovery and a high preconcentration factor. Many adsorbents such as activated carbons, nano sulfur, composite materials (CM), mesoporous silicate materials, and mesoporous silicate nanoparticles (MSN) have been successfully used for this purpose.

Since 1992, mesoporous silicate materials, especially MCM-41, have been received growing attention because of their unique properties such as high surface area and tunable pore size which facilitate the diffusion of large reactants and products inside the pores [32, 33]. These properties allow easy access of functional groups (R = OH, CN, S, NH_2_) into the structure, which increases the adsorption capacity due to the combination of chemical and physical adsorption processes [34]. The mesoporous silicate nanoparticles (MSN) have higher surface area and therefore higher physical adsorption than mesoporous silicate materials [35]. The properties of MSN have been investigated by many research groups in many fields such as catalysis [35], separation [36], optic [37], biotechnology [38–40], and selective adsorbents. These nanomaterials are widely used as adsorbents for adsorption of heavy metal ions from aqueous solution in the field of water treatment [41, 42]. UVM-7 is an interesting material which can be considered as a nanometric version of the well-known MCM-41 material, and the most outstanding feature of this material is its peculiar architecture: a continuous network constructed from aggregates formed from connected mesoporous silicate nanoparticles in the range of meso/macropores, and so it has a bimodal pore system [43].

In this study, UVM-7 as bimodal mesoporous nanoparticle was synthesized and subsequently functionalized with amino silane via grafting method. The main purpose of the present research is to develop a novel analytical method for speciation and preconcentration of trace amounts of Cr (VI)/Cr (III) ions in water samples without the application of any chelating agents. The method is based on the combination of NH_2_-UVM7-packed micro-column SPE and F-AAS detection method. To the best of our knowledge, no attempt has been made to apply NH_2_-UVM-7 in SPE procedure for preconcentration of heavy metal ions, especially for speciation of Cr ions, in water samples. All main factors for the quantitative recoveries of Cr ions were investigated and optimized. The developed method was successfully applied for speciation and determination of analytes in real water samples. Also, the synthesized adsorbent can be used for removal of Cr (VI) ions from water samples.

## Methods

### Apparatus and materials

The determination of chromium ions was performed using spectra GBC flame atomic absorption spectrometer (F-AAS, GBC, Plus 932, Australia) under the conditions given by the manufacturer with air-acetylene flame and deuterium background correction. A Cr hollow cathode lamp operating at a current of 6 mA and a wavelength of 357.9 nm with a spectral bandwidth of 0.2 nm was used. The instrumental conditions were listed in Table 1. The pH values were measured with a Metrohm pH-meter (model 744, Herisau, Switzerland). A peristaltic pump (Lambda, Switzerland) was used in the SPE process.

**Table 1:** Instrumental conditions for chromium determination by F-AAS

Parameters	F-AAS
Wavelength	357.9 nm
Slit	0.2 nm
Lamp curent	6 mA
Injection mode	Manual
Volume Injection	2.2 mL/min
Mode	Integration
Fuel	Air-Acetylene

All chemical compounds and reagents used for experiments and analysis were of analytical grade and purchased from Merck (Darmstadt, Germany). Cr (III) stock solution was prepared by dissolving of appropriate amount of Cr (NO_3_)_3_.9H_2_O (Merck,) as 1000 mg L^−1^ solution in 1 % HNO_3_. Cr (VI) stock solution was prepared by dissolving of appropriate amount of K_2_Cr_2_O_7_ (Merck) as 1000 mg L^−1^ solution in 1 % HCl. Standard and experimental solutions were prepared daily by dilution of the stock solution. The pH adjustments of samples were made using nitric acid (0.1 mol/L) for pH 1-2, and appropriate buffer solutions including sodium acetate (CH_3_COONa/CH_3_COOH, 1-2 mol/L) for pH 3-7, and ammonium chloride (NH_3_/NH_4_Cl, 0.2 mol/L) for pH 8-10. Deionized water obtained from a Millipore Continental Water System (Bedford, MA, USA) was used throughout this study. All the laboratory glassware and plastics were cleaned by soaking in 10 % (v/v) nitric acid for at least 24 h and then rinsed with deionized water prior to use.

### Synthesis and functionalization of UVM-7

The general procedure for synthesis of UVM-7 is the atrane route, in which the presence of the polyalcohol is the key to balancing the hydrolysis and condensation reaction rates. In a typical synthesis, TEOS (tetraethyl ortho-silicate) was added to predetermine amounts of TEAH_3_ (triethanolamine). The solution was heated up to 140 °C under vigorous stirring. After cooling down to 90 °C, CTAB (cetyltrimethylammonium bromide) was added to this solution. Hereafter, water was added slowly to the solution under stirring until a white suspension was obtained. This suspension was kept for 4 h at room temperature. The solid was filtered, washed with sufficient amounts of water and acetone and dried in an oven at 80 °C overnight. Thermocalcination of the as-synthesized UVM-7 was carried out under flow of air up to 550 °C for 6 h with a heating rate of 1 °C/min to remove both the surfactant and TEAH_3_ from the as-synthesized UVM-7. The final molar composition of the reactants was 1.0 TEOS: 3.5 TEAH_3_: 0.25 CTAB: 90 H_2_O.

For the functionalization of calcined UVM-7 with amine groups, 1.2 g of triethoxysililpropylamine (C_9_H_23_NO_3_Si) and 2 g of calcined UVM-7 were added to appropriate amount of toluene and refluxed for 24 h at 80 °C, followed by filtering and washing with proper amounts of ethanol and deionized water. The obtained bulky amine-functionalized UVM-7 was dried at 80 °C overnight.

### Characterization of adsorbent

X-ray diffraction (XRD) patterns were recorded on a Seifert TT 3000 diffractometer (Ahrensburg, Germany) using nickel filtered Cu-Kα radiation of wavelength 0.15405 nm. The textural properties of the sorbent including surface area, pore volume, and pore size distribution were determined by nitrogen adsorption–desorption isotherms at-196 °C using BELSORP-mini porosimeter (Bell Japan, Inc.). Prior to analysis the samples were degassed under vacuum at 300 °C for 4 h until a stable vacuum of 0.1 Pa was reached. The specific surface areas and pore volume of the sorbents were calculated by the Brunauer-Emmett-Teller (BET) and Barrett-Joyner-Halenda (BJH) methods, respectively. Scanning electron microscopy (SEM, Phillips, PW3710, Netherland) was used for surface image analysis of the sorbents. The morphology of sorbent was examined by transmission electron microscopy (TEM, CM30, Philips, Netherland). The elemental analyzer (CHNS/O, PerkinElmer, 2400 Series II, USA) was used for determination of elemental composition of sample. CHN instrument performs elemental ratio calculations of H/C, N/C, S/C or C/N.

### General procedure

In SPE procedure, thin wall polymer tubing with outer diameter of 0.55 cm, inner diameter of 0.5 cm, length of 5 cm, and wall thickness of 0.05 cm filled by bulky nanoparticles of NH_2_-UVM-7 was used as a preconcentration micro-column. An aliquot of the sample solution (100 mL) containing 20 μg/L of Cr (VI) and/or Cr (III) was adjusted to pH = 2 with nitric acid solution (0.1 N). The resulting sample solution was pumped through the NH_2_-UVM-7 packed micro-column at a flow rate of 2.5 mL/min. The micro-column was then rinsed with 5 mL deionized water. Afterwards, the retained Cr (VI) ions were completely eluted from the solid phase with 1.5 mL of 0.3 mol/L NaOH solution. Finally, the concentration of Cr (VI) ions in the eluent was determined by F-AAS. The recovery was calculated by using Eq. 1, where *C*_*i*_ is the initial concentrations of analytes in solution phase, and *C*_*f*_ is the concentration of analytes determined by F-AAS after the SPE process. All the experimental data were the averages of triplicate determinations.

1 $$ \mathrm{Recovery}\;\%=\frac{\left({\mathrm{C}}_{\mathrm{i}}\hbox{-} {\mathrm{C}}_{\mathrm{f}}\right)}{\mathrm{C}\mathrm{i}}\times 100 $$

In the optimum pH conditions, Cr (VI) ions were complexed with amine groups of NH_2_-UVM-7 and total chromium was calculated by oxidation of Cr (III) to Cr (VI) with H_2_O_2_ solution (1 mL, 0.2 M). Then, Cr (III) concentration of water sample was simply calculated by the difference between total chromium and Cr (VI) concentrations. A blank solution was also run under the same analytical conditions without adding any chromium ions. The NH_2_-UVM-7 adsorbent was used freshly for blank experimental run. Moreover, the same procedure was repeated for unmodified UVM-7 sorbent. The extraction conditions were listed in Table 2.

**Table 2:** Extraction Conditions of proposed method for chromium speciation

Parameter	Value
Working pH	2
Amount of NH_2_-UVM-7	0.12 g
Sample volume of SPE	100 mL
Volume of sample injection	1.5 mL
Linear range (method)	6-320 μg/L
Limit of detection (LOD)	1.2 μg/L
Preconcentration factor (PF)	66.7
Buffer concentration	0.03 mol/L
Volume of back-extraction solvent (NaOH)	1.5 mL
Concentration of back-extraction solvent (NaOH)	0.3 mol/L
Linear range (F-AAS)	0.4 - 15 mg/L
Correlation coefficient of F-AAS	R^2^ = 0.9956

## Results

### XRD analysis

**Fig. 1:**
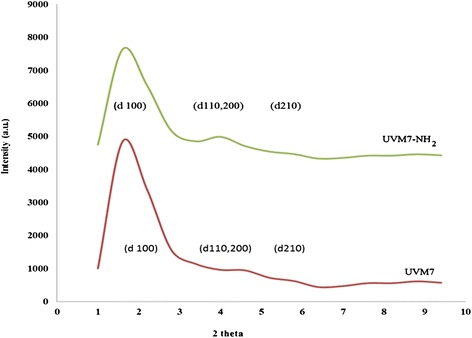
Low angle XRD patterns of calcined NH_2_-UVM-7 and UVM-7

XRD patterns of calcined UVM-7 and amine-grafted UVM-7 are shown in Fig. 1. There are three resolved diffraction peaks in XRD patterns of NH_2_-UVM-7 and UVM-7, which can be indexed as the (100), (110), (200) and (210) reflections associated with hexagonal symmetry (d_110_ and d_200_ were overlapped with each other). However, these peaks are broad, which is the characteristic of mesoporous materials synthesized via atrane route. After the attachment of organic groups on the silica wall of UVM-7, the main three diffraction peaks are still clear which means that functionalization procedure did not had worth effect on the structural order of UVM-7.

### N_2_ physisorption

**Fig. 2:**
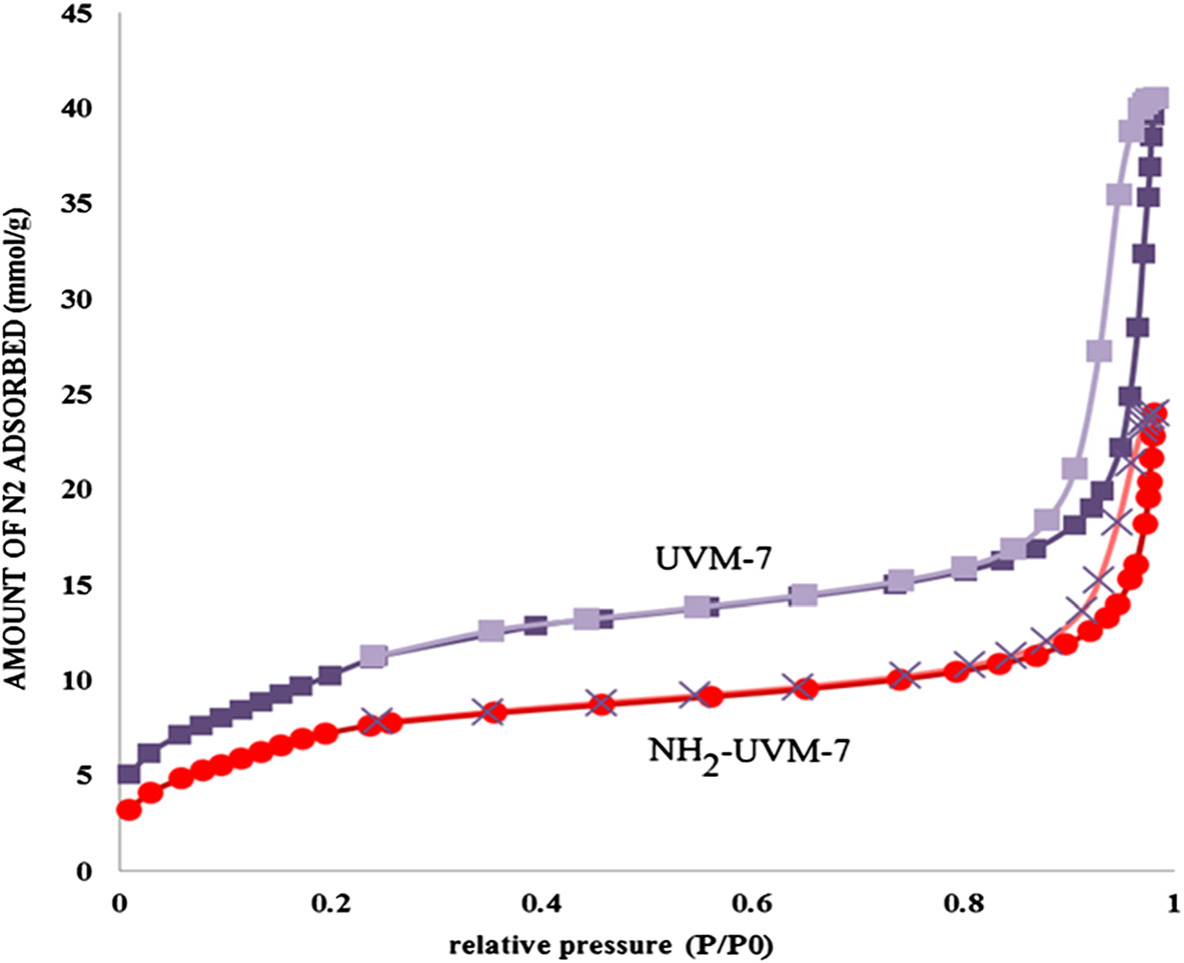
Nitrogen physisorption isotherms of NH_2_-UVM-7 and UVM-7

The nitrogen adsorption-desorption isotherms of UVM-7 and NH_2_-UVM-7 were determined and displayed in Fig. 2. The corresponding isotherm of both samples displays two distinct regions at medium and at high relative pressure which can be attributed to the presence of bimodal pore system. The first is related to the presence of small mesopores (IUPAC clacification), and the second is related to the large mesopores, respectively. The observation of these two distinct regions in both the samples, UVM-7 and NH_2_-UVM-7, confirms that the UVM-7 bimodal pore system is remained almost intact after the functionalization with triethoxysililpropylamine. Textural properties of UVM-7 and NH2-UVM-7 were determined and presented in Table 3. The specific surface area (S_BET_) of UVM-7 and NH_2_-UVM-7 calculated from the linear part of the BET equation were 863 m^2^/g and 626 m^2^/g, respectively. Decreasing of BET surface area, pore volume, and pore diameter of NH_2_-UVM-7 in comparison with initial UVM-7 is due to the grafting of aminosilane on silica walls. The unit cell parameter (a_0_) and the average pore wall thickness (W_t_) of the sorbents were calculated by the equations of a_0_ = 2.d_100_/√3 and W_t_ = a_0_– (d_p_/1.05), respectively, where d_p_ is the pore diameter of adsorbent and d_100_ is obtained from XRD diffractograms. As shown in Table 3, these two parameters are almost constant for both the sorbents.

**Table 3:** Textural properties of UVM-7 and NH_2_-UVM-7

Sample	S_BET_^a^(m^2^/g)	d_sp_^b^(nm)	d_lp_^c^(nm)	V_sp_^d^(cm^3^/g)	V_lp_^e^(cm^3^/g)	a^f^(nm)	W_t_^g^(nm)
UVM-7	863	2.67	52.2	0.42	0.84	5.59	2.92
NH_2_-UVM-7	626	2.62	42.2	0.27	0.41	5.59	2.97

^a^BET specific surface area, ^b^diameter of small pores, ^c^diameter of large pores, ^d^Volume of small pores, ^e^Volume of large pores, ^f^Unit cell parameter obtained from XRD diffractograms (2*d*_100_/√3), ^g^Wall thickness(nm) obtained by following equation: W_t_ = a– (d_p_/1.05)

### Elemental analysis

Elemental analysis provides further evidence for the amount of amine functional groups grafteed on UVM-7. The yield of functionalization can be calculated using the elemental analysis results (the ratio of nitrogen content of the amine-functionalized UVM-7 divided by the amount of nitrogen of the triethoxysililpropylamine used for functionalization). The nitrogen content was 4.83 wt %. Moreover, by determining the nitrogen content of the remaining toluene it was found that about 99 % of the triethoxysililpropylamine was grafted on the UVM-7.

### SEM imaging

**Fig. 3:**
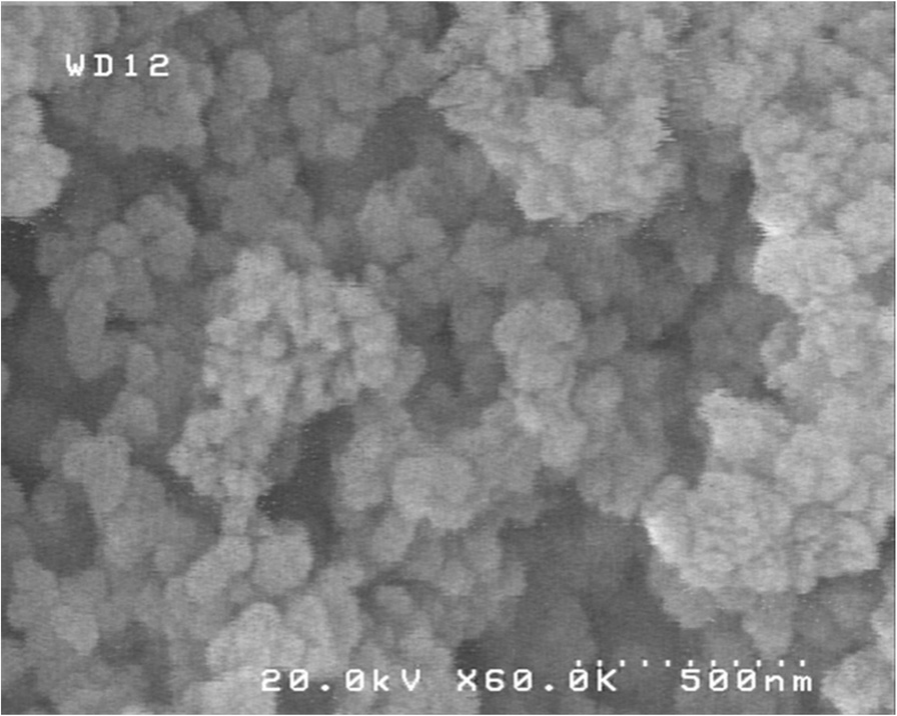
SEM image of NH_2_-UVM-7

The SEM was performed to illustrate the morphology and particle size distribution of the calcined NH_2_-UVM-7. As shown in Fig. 3, NH_2_-UVM-7 has a highly porous morphology and the mesoporous silica particles are in nanometer range (30 nm). Moreover, functionalization did not led to bulky silica nanoparticles.

### TEM imaging

**Fig. 4:**
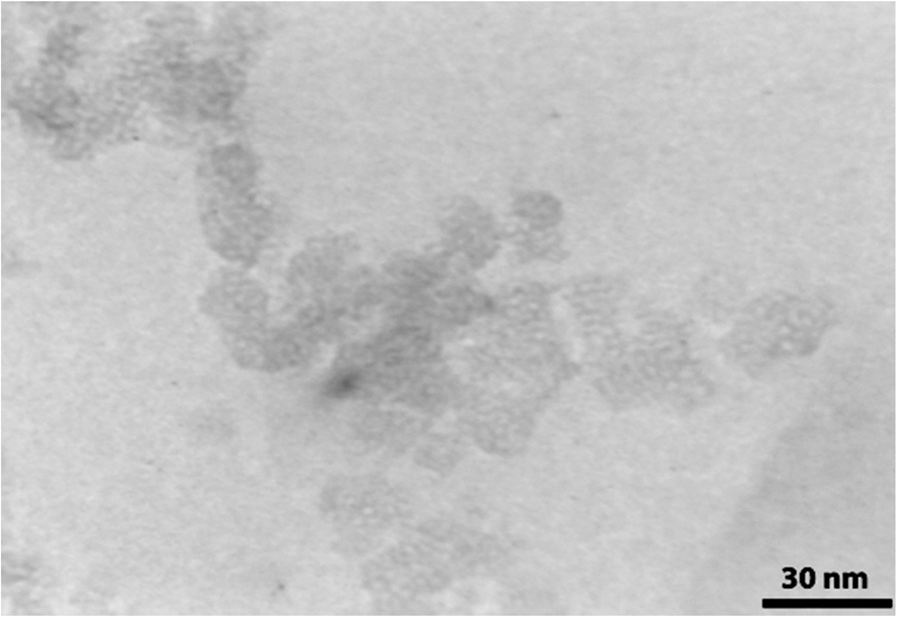
TEM image of NH_2_-UVM-7

TEM image also illustrates pore structure of NH_2_-UVM-7. As shown in Fig. 4, the mesopores are clearly visible in the silica nanoparticles and particle size of the samples is in nanometer range around 30 to 40 nm as those observed in SEM image.

### Effect of pH, sample volume and amount of NH_2_-UVM-7

**Fig. 5:**
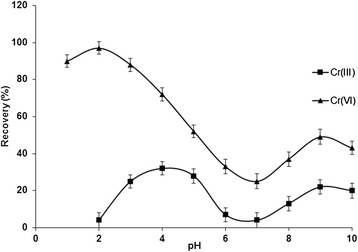
The influence of solution pH on the recovery of Cr (**VI**) (▲) and Cr (**III**) (■) ions with NH_2_-UVM-7 (▲). Conditions: sample volume 100 mL; adsorbent amount 0.12 g; eluent 1.5 mL of 0.3 mol/L NaOH; sample flow rate 2.5 mL/min

In the SPE studies, the pH of the sample solution is an important parameter to obtain quantitative recoveries of metal ions, because it affects the surface charge of the adsorbent, the degree of ionization and speciation of the adsorbate. The influence of sample pH on speciation and recovery efficiency of Cr (VI)/Cr (III) ions by NH_2_-UVM-7 was investigated at different pH values (1-10) by using buffered sample solutions containing Cr (III) and Cr (VI) ions, according to the general procedure. The complexation was strongly conditioned by the pH of solutions and subsequently affects extraction efficiency of the complex. As shown in Fig. 5, the highest extraction efficiency (>97 %) for Cr (VI) was achieved in the pH range of 1 -3.2, whereas the recovery values for Cr (III) were below 5 % in the same pH. Thus, the procedure was applied to speciation of Cr (VI)/Cr (III) ions at pH = 2. For higher pH values (pH > 3), the recovery percentages of Cr (VI) ions decreased with increase in pH. The extraction mechanism of Cr (VI) ions on the NH_2_-UVM-7 may be based on the electrostatic attractions between metals ions and NH_2_ groups of nanoadsorbent, which is highly dependent on solution pH. The NH_2_ groups on the surface of the NH_2_-UVM-7 can either be protonated (-NH_3_^+^) at low pH or be deprotonated (NH^−^) at high pH. The variation in adsorption capacity of Cr (VI) at different pH values may be attributed to the affinities of NH_2_-UVM-7 nanoadsorbent for the different anionic species of Cr (VI) existing at acidic pH conditions namely HCrO_4_^−^, CrO_4_^2−^ and Cr_2_O_7_^2−^. It is clear that negatively charged HCrO_4_^−^ and Cr_2_O_7_^2−^ are easily adsorbed to the positively charged NH_3_^+^ at low pH values due to the electronic attraction. By using UVM-7 as the SPE adsorbent for preconcentration of Cr (IV) ions, it was observed that the extraction efficiencies were less than 5 % in the studied pH range. This is because the physical adsorption of Cr (VI) ions by unmodified UVM-7 is the main mechanism. So, the NH_2_-UVM7 is favorite adsorbent for speciation and determination of Cr (VI)/Cr (III) ions in water samples by SPE method and can be used for on-line removal Cr (VI) in water samples at pH = 2.

**Fig. 6:**
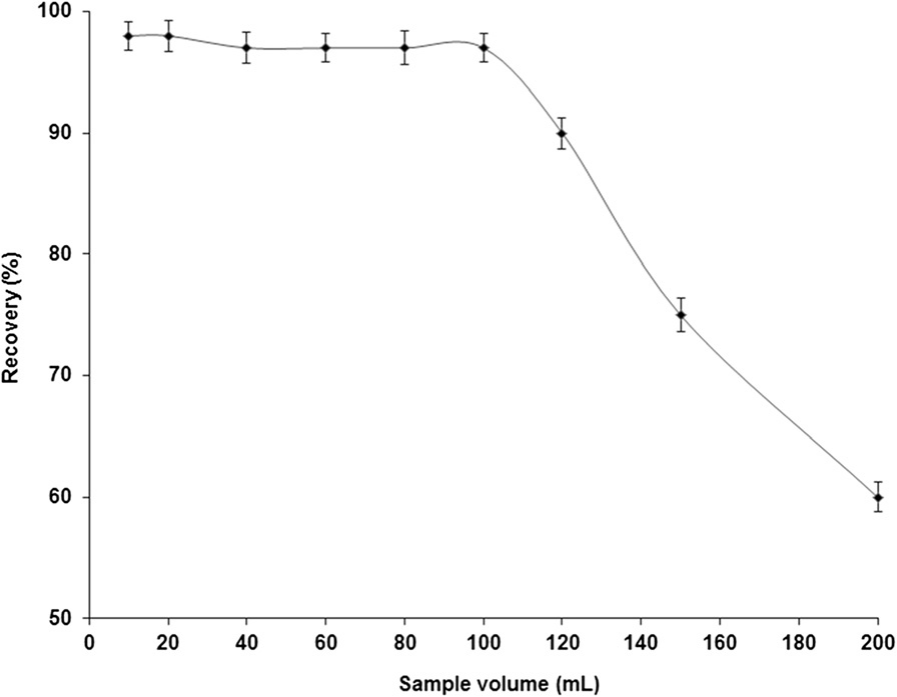
The influence of sample volume on the recovery of Cr (**VI**) ions. Conditions: solution pH 2; adsorbent amount 0.12 g; eluent 1.5 mL of 0.3 mol/L NaOH; sample flow rate 2.5 mL/min

Sample volume is one of the most important parameters of SPE procedure. The effect of aqueous sample volume on the recoveries of analyte ions was examined by passing different volumes (10-200 mL) of sample solution from the micro-column, according to the general procedure. The results were illustrated in Fig. 6. Quantitative recoveries of Cr (VI) ions were obtained using 10-100 mL of sample solution. At higher volumes, the recoveries were decreased. Therefore, 100 mL was selected as the optimum sample volume for further experiments of SPE. The preconcentration factor (PF) for preconcentration and extraction is calculated by the ratio of the highest sample volume for analyte (100 mL) and the lowest final eluent volume (1.5 mL). In the present study the possible preconcentration factor was 66.7.

**Fig. 7:**
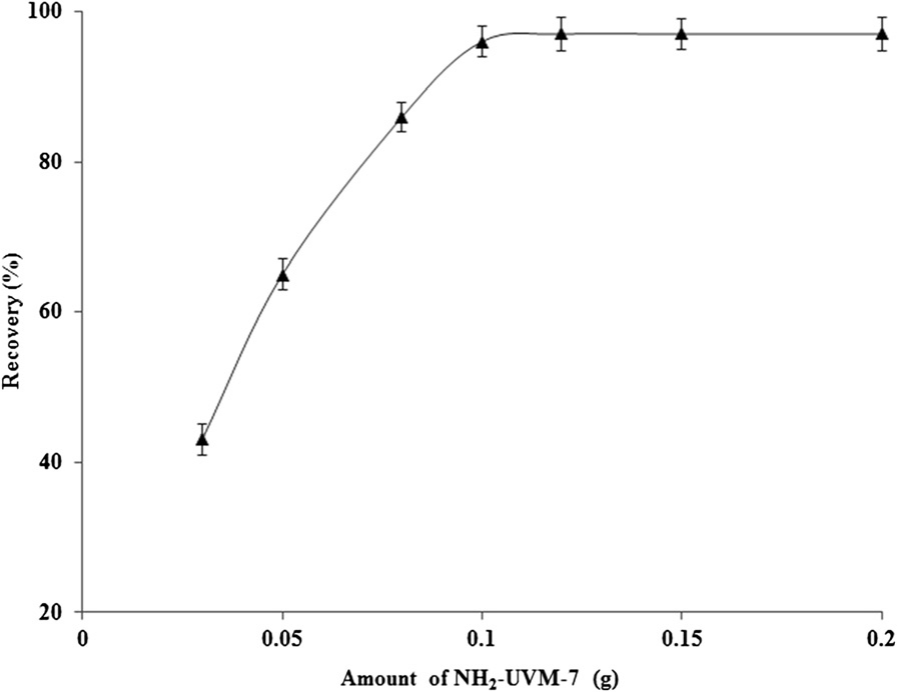
The influence of amount of NH_2_-UVM-7 on the recovery of Cr (**VI**) ions. Conditions: sample volume 100 mL; solution pH 2; eluent 1.5 mL of 0.3 mol/L NaOH; sample flow rate 2.5 mL/min

The effect of the adsorbent dosage on the recoveries of Cr (VI) ions was investigated using various amounts of NH_2_-UVM-7 in the range of 0.03–0.2 g (Fig. 7). It was observed that the extraction efficiency of the system was remarkably affected by NH_2_-UVM-7 amount, and quantitative extraction was obtained using 0.1-0.2 g of NH_2_-UVM-7. Therefore, in order to achieve a suitable preconcentration, 0.12 g of NH_2_-UVM-7 was chosen as optimum leading to a final adsorbent.

### Effect of matrix and various eluents

In this section, the interfering effect of various matrix ions, most probably present in the environmental samples, on the recoveries of Cr (VI) ions from NH_2_-UVM-7 based SPE was examined. The procedure of SPE was performed using a 100 ml sample containing 100 μg/L of analyte and 1–2 mg/L of different concentration of matrix ions. The tolerate amounts of each ion were the concentration values tested that caused less than 5 % the absorbance alteration. As shown in Table 4, the ions normally present in natural waters has no significant influence on the adsorption of Cr (VI) ions under the experimental conditions used.

**Table 4:** The effect of matrix ions on chromium speciation and determination by proposed method

Ions	Maximum tolerance ratio (matrix ion conc./Cr conc.)
Ni^2+^, Mg^2+^, Ca^2+^, Co^2+^, Al^3+^, K^+^, Na^+^,	2000
V^3+^, Mn^2+^, Fe^3+^, Pb^2+^, Zn^2+^	
PO_4_^3−^, Cl ^-^, CO_3_^2−^, SO_4_^2−^, NO_3_^−^	1000

This work was performed using 100 mL of 100 μg/L Cr (VI) standard solution

**Fig. 8:**
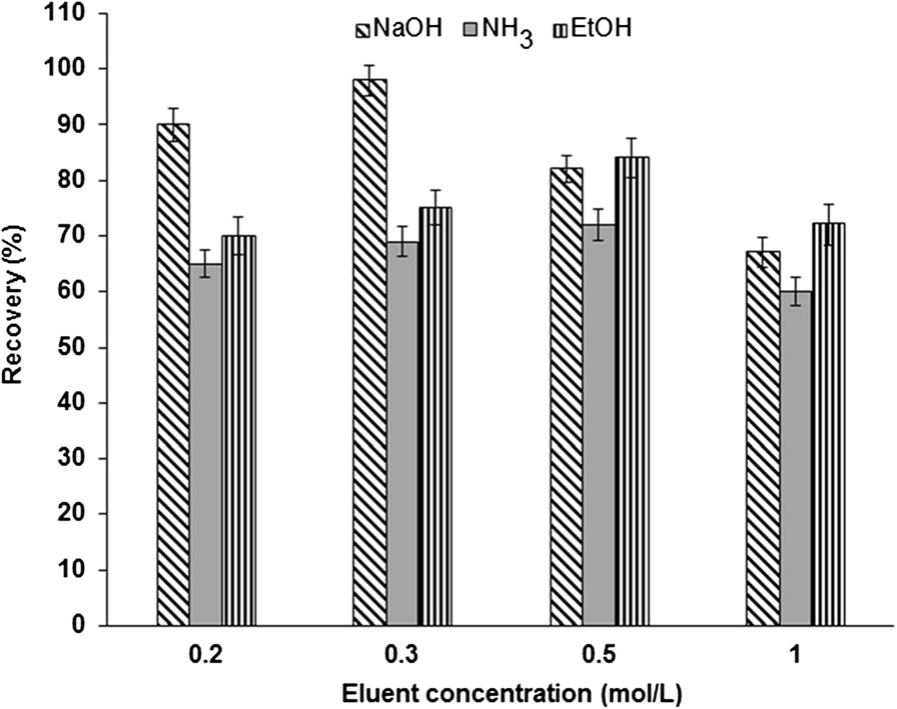
Effect of different eluents on the recovery of Cr (**VI**) ions from NH_2_-UVM-7 phase. Conditions: sample volume 100 mL; solution pH 2; adsorbent amount 0.12 g; sample flow rate 2.5 mL/min; eluent volume 1.5 mL

The proposed method was based on back-extraction of Cr (VI) ions from NH_2_-UVM-7 with a suitable basic elution solution. Increasing of the pH leads to dissociation and releasing of chromium ions into the aqueous phase. A volume of 1.5 mL of various eluents such as NaOH, NH_3_, and EtOH (ethanol) with different concentration of 0.2-1 mol/L were tested as the eluent. The results showed that 1.5 mL of 0.3 mol/L NaOH quantitatively extracted chromium ions from the solid phase. At high concentration of NaOH, the recovery percentage was decreased (Fig. 8).

### Method validation

The developed method was applied for speciation/preconcentration and determination of Cr (VI) and Cr (III) ions in different water samples including Tap and drinking water (Tehran, Iran), river water (Kan, Tehran, Iran), waste water (petrochemical factory, Tehran, Iran) and industrial water (Tehran, Iran). The spiked samples were prepared to demonstrate the accuracy and reliability of the method for determination of Cr (III) and Cr (VI). The remaining aliquots were spiked with increasing quantities of Cr (VI) and Cr (III) and analyzed by the developed SPE method. As it can be seen from Table 5, a good agreement was obtained between the added and measured analytes amount, which confirms the accuracy of the procedure and its independence from the matrix effects. The recovery of spiked samples is satisfactorily reasonable and was confirmed using addition method, which indicates the capability of the system in the determination of Cr (VI) and Cr (III) in water and biological samples. The validation of the presented procedure was also performed by the analysis of certified reference material, NIST SRM 1643e (Trace elements in water), from the National Institute of Standard and Technology (NIST, Gaithersburg, USA), for chromium values. The results found were in good agreement with the certified values of SRM (Table 6).

**Table 5:** The reliability of proposed method for determination and speciation of Cr (III) and Cr (VI) in natural water samples

Sample	Added (μg/L)		Found (μg/L)^a^		Total Cr ^a^	Recovery (%)	
	Cr^3+^	Cr^6+^	Cr^3+^	Cr^6+^		Cr^3+^	Cr^6+^
Tap water	---	---	22.8 ± 1.2	ND^b^	22.8 ± 1.2	---	---
	50	---	71. 5 ± 3.4	ND	71.5 ± 3.4	97.4	---
	---	20	23.1 ± 1.8	19.5 ± 1.1	42.6 ± 2.3	---	97.5
Drinking water	---	---	8.7 ± 0.4	11.2 ± 0.5	19.9 ± 0.7	---	---
	50	---	57.9 ± 2.8	10.8 ± 0.4	68.7 ± 3.2	98.4	---
	---	20	8.4 ± 0.3	30.5 ± 1.3	38.9 ± 1.6	---	96.5
Waste water ^c^	---	---	94.6 ± 4.8	52.3 ± 3.1	146.9 ± 6.8	---	---
	50	---	145.4 ± 6.5	50.1 ± 2.7	195.5 ± 9.6	101.6	---
	---	50	93.3 ± 4.3	100.6 ± 5.4	193.9 ± 8.5	---	96.6
River	---	---	17.6 ± 0. 8	9.4 ± 0.3	27.0 ± 1.4	---	---
	10	---	27.3 ± 1.3	9.6 ± 0.4	36.9 ± 1.9	97.0	---
	---	20	17.9 ± 0.7	28.9 ± 1.6	46.8 ± 2.4	---	97.5
Industrial water	---	---	49.1 ± 2.2	125.3 ± 5.1	174.4 ± 9.1	---	---
	40	---	88.6 ± 4.2	122.7 ± 4.8	211.3 ± 9.8	98.8	---
	---	40	48.8 ± 2.2	163.8 ± 6.7	212.6 ± 11.2	---	96.3

^a^Mean of three determinations ± confidence interval (P = 0.95, n =5), ^b^Not Detected, ^c^ from petrochemical factory

**Table 6:** Validation of proposed method for chromium determination by certified reference material (N = 5)

Bovine serum	Certified (μg/L)	Found^a^ (μg/L)	Recovery (%)
NIST SRM 1643e	20.40 ± 0.24	19.48 ± 0.87	95.49 ± 0.44

^a^Mean value ± ts/√N, SRM 1643e consists of approximately 250 mL of acidified water in a polyethylene bottle

### Adsorption capacity

The adsorption capacity of NH_2_-UVM-7 was determined for Cr (VI) by using column technique. 100 mL of aqueous solutions containing 5-500 mg/L Cr (VI) at optimized pH were passed through a micro-column filled with 0.12 g of NH_2_-UVM-7 at a flow rate of 0.5-5 ml/min. After Cr (VI) was eluted from the nanosorbent with 1.5 mL of NaOH (0.3 mol/L), the concentration of Cr (VI) in eluent was determined by F-ASS. In order to successfully represent the dynamic adsorptive behaviour, it is important to have a satisfactory description of the equation state between the two phases composing the adsorption system. In this study the Langmuir adsorption isotherm was employed for Cr (VI) adsorption by NH_2_-UVM-7 adsorbent. The Langmuir model assumes uniform energies of adsorption onto the surface of NH_2_-UVM-7. The Langmuir equation is defined as Eq. 2:

2 $$ \frac{{\mathrm{C}}_{\mathrm{e}}}{{\mathrm{q}}_{\mathrm{e}}}=\frac{1}{{\mathrm{bQ}}_{\kern0.1em  \max }}+\frac{{\mathrm{C}}_{\mathrm{e}}}{{\mathrm{Q}}_{\max }} $$

**Fig. 9:**
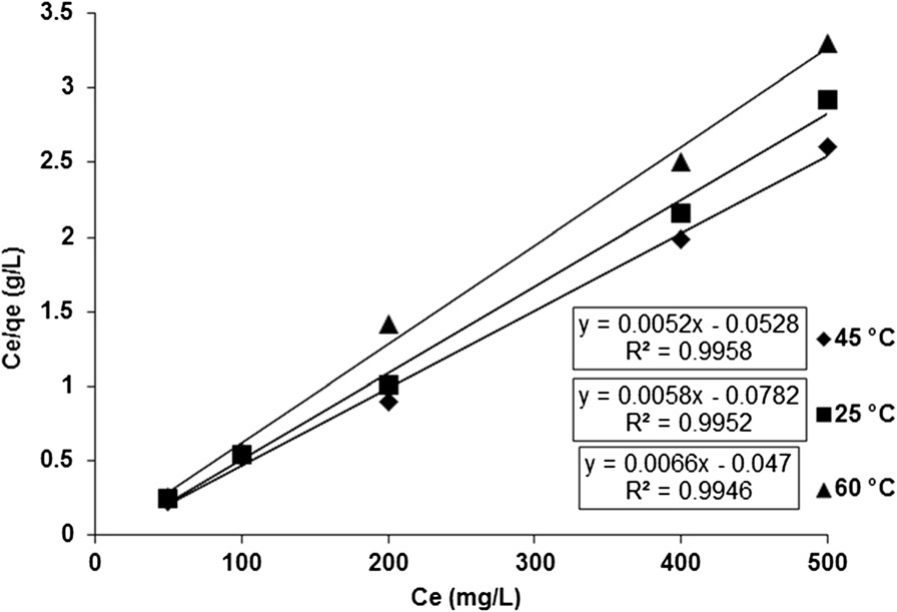
Langmuir plot for chromium adsorption by NH_2_-UVM-7 at different temperatures

Where *C*_*e*_ is the equilibrium concentration of metal ions in solution phase (mg/L) and *q*_*e*_ is the amount of metal ions adsorbed at equilibrium (mg/g), *Q*_max_ is maximum adsorption capacity (mg/g) on unit mass of adsorbent, and b is the Langmuir constant (L/mg), related to the free energy of adsorption. The Langmuir model provided a good fit throughout the concentration range. The applicability of the isotherm models and the high values of the correlation coefficients (R^2^ = 0.9958) for Cr (VI) suggest favourable adsorption by NH_2_-UVM7 at 45 °C. The value of *Q*_*max*_ in Langmuir plots was 192 mg/g for Cr (VI) ions at 45 °C. The values of *Q*_*max*_ and effective time for flow rate are depended on temperature of solution. According to the results, the maximum capacity of adsorbent in column condition due to the shorter contact time is 5 % less than batch system, which is apparently quite expected. The Langmuir isotherm plots at different temperatures and the value of model constants were shown in Fig. 9 and Table 7, respectively.

**Table 7:** The Langmuir isotherm parameters for Cr (VI) adsorption by NH_2_-UVM-7 at different temperatures

Temperature(°C)	Langmuir isotherm			
	Q_max_(mg/g)	Max C_e_/q_e_	R^2^	t_effective_(min)
25	172.4	2.61	0.9952	100
45	192.3	2.92	0.9958	88
60	151.5	3.31	0.9946	83

## Discussion

The results showed that the quantitative recovery of Cr (VI) ions was obtained using 12 mg of NH_2_-UVM-7 sorbent at pH = 2. At sample pH of 3.5-5, Cr (III) ions physically adsorbed on UVM-7 and NH_2_-UVM-7 sorbents with recovery percentages of 33 % and 29 %, respectively. The NH_2_ groups functionalized on UVM-7 were used for speciation of chromium ions at optimized pH, but they were decomposed by using strong acid or base. For back extraction of retained Cr (VI) from NH_2_-UVM-7, the concentration of sodium hydroxide must be less than 0.5 mol/L. However, the pH value of the sample should be adjusted according to the chemistry of the compound of interest. It seems that, at the optimum pH the Cr (VI) ions are in the anionic form and so easily complexed with protonated amine groups (NH_3_^+^) of functionalized UVM-7. Therefore, by controlling pH and amount of sorbent, Cr (VI) ions can be removed from waters. So, the NH_2_-UVM-7 packed micro-column SPE is a favorite method for speciation and determination of chromium ions in environmental samples, and also can be used for on-line removal of Cr (VI) in water samples at optimum pH of 2. Because of high surface area of nanosorbent (S/V) a very little amount of NH_2_-UVM-7 (0.12 g) was used for removal of Cr (VI) in water sample with good adsorption capacity as 192 mg/g. Understanding the chemistry of the compound with analysis such as ionizability and hydrophobicity can be useful in designing appropriate conditions to obtain efficient extraction recovery. The results of this study showed that the sodium hydroxide solution (0.3 mol/L) was more efficient eluent for Cr (VI) back extraction from the sorbent (Fig. 8). The NaOH solution is organic free eluent and can prevent co-elution of organic compounds possibly present in the real samples. In the SPE, the volume required to elute the Cr (VI) from the nanosorbent, depends on the sorbent mass and strength of Cr (VI) retention by NH_2_-UVM-7. In this study, the lowest satisfactory eluent volume is 1.5 mL, giving a suitable preconcentration factor of 66.7. Using this volume, efficient recovery of 97 % can be achieved. In optimized sample volume and sample flow rate less than 5 ml/min, Cr (VI) extraction is applicable with an efficient recovery. In this study sample flow rates up to 3 ml/min were applied with acceptable recovery of 97 %. Therefore, to be confident, the sample flow rate of 2.5 ml/min was selected, providing a reduced extraction time for as large as 100 mL sample volume. However, as the results shows, it would be possible to increase the sample flow rate even more as 4 mL/min without significant loss in the analyte recovery. In order to show the effect of possible matrix components on the developed method, the interfering effects of various matrix ions, most probably present in the environmental samples were examined (Table 4). The ions added to the samples are mostly present in the real water samples and can be used as closely related interferences present in matrices. The results clearly show the non-effectiveness of the all added components for each concentration on the recoveries obtained from developed method. Many sample preparation methods were used for metal extraction. Recently, liquid-liquid extraction and liquid-liquid micro-extraction (LLE, LLME) methods have been used for some heavy metals [44]. Although the LLME technique may be useful in some conditions, however the analyte extraction is too hard as such solutions are extremely difficult to be separated. Therefore, due to such problems, nowadays, there is a strong trend towards replacing LLE by SPE. Based on reported methods for optimizing of SPE [45, 46], authors generally have used 5 factors to optimize the method for environmental samples while, in this study, 8 parameters were screened. This allows that a robust and more reliable method is introduced for environmental monitoring of Cr (VI) and Cr (III) ions. Therefore, to make an advantage from this study compare to the other, further experiments of reproducibility of the method were carried out on spiked water samples to validate the possible use of the developed SPE for determination of Cr (VI) and Cr (III) ions. A comparison of the current method with the other reported methods was also given in Tables 8 and 9. It can be seen that the PF and LOD values of the present method, for determination of trace chromium ions, are better than, or comparable to that reported in the literature [47–56]. The limit of detection, preconcentration factor and adsorption capacity were 1.2 μg/L, 66.7, and 192 mg/g, respectively. Lower LOD values of some other works are related with higher sensitivity of the instrument used in these studies (Table 8). Also, the low sample volume (100 mL) caused to get relatively low preconcentration factor of 66.7. The adsorption of chromium ions on the most adsorbent used in SPE techniques requires formation of hydrophobic complexes using chelating agents [52–54]. However, in the developed method, NH_2_-UVM-7 was investigated as a novel sorbent for the speciation and preconcentration of trace amounts of Cr (VI) and Cr (III) ions without application of any chelating agents. Hazer and Demir used SPE method based on Poly 1, 3-thiazol-2-yl methacrylamide-co-4-vinyl pyridine-co-divinylbenzene for the speciation analysis of chromium. The limit of detection, pre-concentration factor and adsorption capacity were obtained as 2.4 μg/L, 30 and 80 mg/g, respectively [56]. Duran et al. used activated carbon from tea-industry wastes (TIWAC) for preconcentration and speciation of chromium without complexing agent, prior to determination by flame atomic absorption spectrometry [55]. The preconcentration factor and adsorption capacity for activated carbon were 50 and 61 mg/g, respectively. Soylak et al. studied on chromium speciation based on coprecipitation of Cr (III) by using praseodymium (III) hydroxide (Pr (OH)_3_) precipitate and neodymium (III) Hydroxide. In their studies, the recovery extraction for Cr (VI), LOD and PF was lower than our developed method [57, 58]. Finally, the developed method based on the combination of NH_2_-UVM-7 packed micro-column SPE and F-AAS detection method was successfully applied for speciation and determination of Cr (III)/Cr (VI) ions in real water samples. In addition, the NH_2_-UVM-7 can be used for removal of Cr (VI) in water samples with high capacity and good repeatability.

**Table 8:** Comparison of the proposed SPE/F-AAS method with various reported procedures for determination of chromium in water samples

Detection method	Separation technique	PF^a^	LOD^b^(μg/L)	Reference
ICP -MS	DLLME^c^	---	0.110	[48]
ET-AAS	SPE^d^	20	0.050	[49]
ET-AAS	BDES^e^	27	0.010	[50]
F-AAS	CPE^f^	58	0.180	[51]
CAdSV^a^	EDCS^g^	---	0.007	[47]
F-AAS	SPE^h^	66.7	1.2	This work

^a^Preconcentration factor, ^b^Limit of detection, ^c^Dispersive liquid- liquid micro extraction, ^d^Solid phase extraction, ^e^Bi-directional electrostacking, ^f^Cloud point extraction, ^g^Electro deposition on cell surface, ^h^Nano solid phase extraction by NH_2_-UVM-7

## Conclusions

The simple, fast, reliable, and economical technique for speciation and determination of trace amount of Cr (VI) and Cr (III) ions in real water samples was developed by combining the SPE technique based on NH_2_-UVM-7 adsorbent with F-AAS detection method. The developed method allows us to obtain good recovery (>97 %), reproducibility, and good preconcentration factor (66.7) without using the chelating agent. In addition, the method was free of interference. Factors influencing the SPE method were optimized. The recovery efficiency of UVM-7 and NH_2_-UVM-7 for Cr (VI) were found to be 5 % and 96 %, respectively, at pH =2. So, the extraction recoveries of Cr (VI) anion are depended on chemically adsorption by protonated amine group (NH_3_^+^) of NH_2_-UVM-7. The developed SPE/F-AAS method based on NH_2_-UVM-7 sorbent was successfully applied for speciation and determination of Cr (VI) and Cr (III) ions in real water samples. This method is comparable respect to powerful techniques as DLLME-ET-AAS (Table 8). The adsorption capacity of NH_2_-UVM-7 for Cr (VI) ions is higher than that chelate modified SPE procedures reported in literature up to now (Table 9). The developed procedure has some advantages over other SPE methods in literature, such as low consumption of only 0.12 g NH_2_-UVM-7 as adsorbent and also 1.5 mL of eluent per extraction, high sorption capacities, low detection limits, good preconcentration factor and good reusability (up to 15 cycles). The developed method can be used for removal of Cr (VI) in water samples at optimized pH. It is expected that the developed method could successfully be utilized for speciation, preconcentration, and determination of chromium ions in different environmental and biological samples.

**Table 9:** Comparison of the proposed method with other SPE procedures for determination of chromium in water samples

SPE adsorbent	LOD^a^ (μg/L)	PF^b^	Adsorption capacity (mg/g)	Reference
DPC-Poly C-18^c^	2.4	12	------	[52]
ENVI-18 DSK ^TM^ disk	20	300	-----	[53]
APDC/MWCNT^d^	0.96	100	9.5	[54]
TIWAC^e^	4.5	50	61.0	[55]
Polymer ^f^	2.4	30	80.2	[56]
NH_2_-UVM-7	1.2	66.7	192.0	This work

^a^Limit of detection, ^b^Pre-concentration factor, ^c^1,5-diphenylcarbohydrazide (DPC)-Polysorb C-18 beads, ^d^Ammonium pyrrolidine dithiocarbamate (APDC)/multi-walled carbon nanotubes, ^e^Tea-industry waste activated carbon, ^f^Poly (1,3-thiazol-2-yl methacrylamide-co-4-vinyl pyridine-co-divinylbenzene)
